# Temperature-induced variation in the transcriptome of maritime pine (*Pinus pinaster* Ait.) embryogenic masses modulates the phenotype of the derived plants

**DOI:** 10.1186/s12864-025-11610-0

**Published:** 2025-05-10

**Authors:** Javier Montero-Pau, María Amparo Pérez-Oliver, Álvaro Rodríguez-Cuesta, Isabel Arrillaga, Ester Sales

**Affiliations:** 1 Institute Cavanilles of Biodiversity and Evolutionary Biology (ICBiBE), University of Valencia, Catedrático José Beltrán Martínez 2, Paterna, 46980 Spain; 2 Biotechnology and Biomedicine Institute (BiotecMed) and Plant Biology Department, Faculty of Pharmacy, University of Valencia, Av. Vicent Andrés Estellés s/n, Burjassot, 46100 Spain; 3 Agrarian and Environmental Sciences Department, Institute for Research on Environmental Sciences (IUCA), University of Zaragoza. High Polytechnic School, Ctra. Cuarte s/n, Huesca, 22197 Spain

**Keywords:** Climate change, Epitypes, Forest-trees, Gene expression, RNA-seq

## Abstract

A number of studies show that combining somatic embryogenesis with environmental stimuli can induce plant defenses against abiotic stresses, offering a complementary strategy in tree breeding programs. In a previous study, we found that increasing/decreasing the standard temperature of 23 ˚C by 5 ˚C during maritime pine *(Pinus pinaster)* somatic embryo maturation resulted in epitypes, as the derived plants showed altered phenotypes regarding leaf histology, proline content, photosynthetic rates, and hormone profiles, and that also differentially respond after a short-term heat stress. To elucidate the mechanisms underlying these altered phenotypes, we sequenced the transcriptome of embryonal-suspensor masses (EMs) from the three epitypes, identifying 812 differentially expressed genes (DEGs). Ten genes involved in epigenetic regulation were specifically up-regulated in EMs of the cold epitype. While some of these genes have been linked with somatic embryo maturation, the increased expression of three of these genes, *histone deacetylases HDA9*, a *histone-lysine methyl-transferase (HKMT)* and an *Argonaute (AGO7)*, was found to be low temperature-induced epigenetic marks. Among the genes up-regulated in the EMs from the warm epitype, we studied those related to abiotic stress response and observed greater variation in genes involved in abscisic acid (ABA)-mediated response such as those encoding Ras GTPase-activating protein-binding (G3BP) proteins, an AAA-ATPase, and an aspartyl protease (APF2). We also found differential expression in genes encoding for RING-type E3 ubiquitin-transferases, and DNAJ and BAG chaperones. Additionally, the biosynthetic pathways of jasmonic acid, cytokinins and the diterpene pimaradiene were also altered in the warm epitype. However, the increased ABA and cytokinin content observed in the plants derived from this warm epitype cannot be fully explained by the EMs transcriptome profile. Conversely, in the cold epitype, we observed downregulation of genes encoding for an ABA receptor (PYL3), and a xyloglucan endotrans-glucosylase/hydrolase (XTH6). These findings support the hypothesis that the previously reported heat-adapted phenotype of plants derived from the cold epitype (characterized by a faster and higher proline increase, lower increases in ABA levels, no reduction in active cytokinins, and a better net photosynthesis rate recovery) could be attributed to low-temperature-induced epigenetic marks that were absent in the warm epitype.

**Supplementary Information:**

The online version contains supplementary material available at 10.1186/s12864-025-11610-0.

## Introduction

Forests in Europe are mostly semi-natural (89%), the rest are covered by plantations. Global warming together with the increase in frequency of drought events in large areas of the Mediterranean, Central and Eastern Europe have caused one out of four trees in EU forests to show defoliation levels indicating damage [1]. Across Europe, tree canopy mortality is accelerating by 1.5% annually, doubling the observed rate in the late 20th century. This is equivalent to 1% of the EU-2 forest area dying yearly [2]. Climate model simulations give a collective picture of a substantial decline in effective rainfall and warming exceeding 4–5 ˚C in the Mediterranean region, especially in the summer [3]. In this context, there is an urgent need for forest-tree breeding programs to deploy superior individuals to improve the resilience of Mediterranean forests. However, breeding programs for conifers, which are among the longest-living organisms with long generation times, cannot rely on classical evolutionary means, such as mutation and natural selection, to respond rapidly to temperature increases driven by global climate change [4].

Among conifers, maritime pine (*Pinus pinaster* Ait.) is one of the most important species in southern Europe, having special ecological and economic relevance since it is often used for timber and resin. The maritime pine is distributed in a wide range of environmental conditions, displaying a high degree of adaptability [5]. Despite its phenotypic plasticity, maritime pine stands show symptoms of decline, mainly defoliation and low natural regeneration, in some areas of the Western Mediterranean [6]. The combination of human use and climate trends, which include increasing temperatures, number of summer days, frequency of drought events, and recurrent fires and pathogen attacks, may influence maritime pine forest health. In fact, competition in unthinned stands and water stress were reported to be major factors accounting for maritime pine decline [7]. The development of improved maritime pine varieties with superior adaptative traits would be useful for afforestation and plantation purposes. For this goal to be achieved, it is important to study how environmental factors during embryo development drive adaptive changes in gene expression in this species.

The role of epigenetic variation contributing to the phenotypic plasticity and adaptative capacity of forest tree species was first reviewed by Bräutigam et al. [8] and more recently by Kurpisz and Pawłowski [9], and Fossdal et al. [10]. Epigenetic marks are modifications of DNA and histones, and/or activity of small RNAs that affect transcriptional activity without changing the DNA sequence. DNA methylation, the most prominent DNA modification, can occur in different sequence contexts and be present at promoters, introns, and transposable elements. The histone N-terminal tails are easily accessed and modified with various covalent modifications, such as methylation, acetylation, ubiquitination, phosphorylation, etc. In turn, N^6^-methyladenosine is the most prevalent chemical modification identified in eukaryotic mRNAs [11]. These reversible modifications induced in gene expression are transmitted through mitosis and eventually also through meiosis [12]. It has been suggested that these modifications together with the genetic component contribute to the phenotypic plasticity and adaptive potential of individuals and populations, therefore modulating plant fitness under environmental stress [8, 13].

Somatic embryogenesis (SE) has demonstrated itself to be a powerful tool for conifer propagation and the study of molecular and physiological mechanisms during embryo development, as well as its influence on environmental constraints. Varying environmental conditions during SE in conifer species can produce epigenetic marks resulting in the formation of epitypes. In line with this, Yakovlev et al. [14] found differences in transcriptomes between the genetically identical Norway spruce embryogenic tissues grown under two epitype-inducing temperatures. These differences accounted for and were explained by chromatin modifications, as some differentially expressed genes encoded for proteins involved in epigenetic machinery that modulate phenotypes [14, 15], and have recently been associated with differences in the methylation of specific genes [16]. More recently, Trontin et al. [17] studied the changes in DNA methylation induced by varying temperature during embryogenesis in a maritime pine somatic embryo genotype and in the shoot apical meristem of its 2-year-old derived somatic plants; these authors identified some genes that might be involved in the establishment of epigenetic marks, therefore suggesting that alterations were transmitted to the generated plants.

Previously, our group demonstrated that by adjusting temperatures during the SE phases (induction, proliferation and maturation), it is possible to improve maritime pine somatic embryo production [18]. We also found that applying heat pulses to the megagametophyte in the initial phase of SE produced plants with better adaptation to short-term heat or drought stresses [19, 20]. In previous studies, following the approach employed by Yakovlev et al. [14] in *Picea abies*, we found that temperature variation (23 ± 5 ˚C) during *Pinus pinaster* somatic embryo maturation resulted in epitypes that generated altered phenotypes in 2-year-old somatic plants, in leaf histology, proline content, photosynthetic rates, and hormone profiles. These plants also respond differentially after short-term heat stress, especially those generated after lowering temperatures during somatic embryo maturation. They were better adapted to stress, indicating a cross-tolerance effect [21]. To establish epigenetic-related environmental stress memory during early embryo development by abiotic stress exposure might induce new traits in conifers that would have considerable practical applications in forest breeding [13]. The potential link between natural epigenetic variation and phenotypic variability observed in trees is further supported by studies in ecotypes and in individual populations of specific herbaceous plant species [8].

The epigenetic and transcriptional regulation of *Pinus pinaster* zygotic embryo development was reported by Vega-Bartol et al. [22], whilst the transcriptome and small RNAs (sRNAs) profiles of developing somatic embryos were reported by Rodrigues et al. [23, 24]. More recently, Ávila et al. [4] described the transcriptome dynamics of zygotic and somatic embryogenesis, identifying metabolic pathways regulated differentially in the two processes. Transcriptomic changes throughout maturation were found to be more pronounced in somatic compared to zygotic embryos. To date, however, few studies have been addressed as to the effect of temperature on the transcriptome of the species. Thus, the objective of our present work was to investigate whether the altered phenotypes observed in maritime pine plants from different epitypes could be explained by transcriptional and epigenetic modifications induced by temperature variation during maturation of maritime pine embryonal-suspensor masses (EMs). Insight into epigenetic variation and its relationship to phenotypic plasticity will contribute to the understanding of adaptive plant responses, and might help to evaluate the risk to conifers of temperature fluctuations in the environment that are relevant in the global warming scenario.

## Materials and methods

### Plant material, RNA extraction and sequencing

Maritime pine EMs were induced using megagametophytes isolated from immature cones sampled in different mother trees (1007, 1046, and 1058) belonging to the Galician Tree Breeding Program (Conselleria do Medio Rural, Xunta de Galicia, Spain). Three EMs lines (1007P3a, 1046P1c and 1058P4c) were established as described in Arrillaga et al. [18], being the induction and proliferation phases performed at 28 ˚C. For maturation, 100 mg aliquots of each line were transferred to maturation medium and incubated at 18, 23 or 28 ˚C as described in Arrillaga et al. [18], producing for each genotype three different epitypes. After 5 weeks in maturation media, 150 mg of each epitype, containing a mixture of EMs and somatic embryos at the precotyledonary stage (Supplementary Fig. 1), were sampled, frozen in liquid nitrogen and stored at -80 ˚C. Total RNA was isolated from frozen samples (9 maturating lines: 3 genotypes × 3 epitypes) using the Plant/Fungi Total RNA Purification Kit (©Norgen Biotek Corp.). Genomic DNA was degraded by using the Recombinant DNase I (RNase-free, Takara Bio Inc., Shiga, Japan), following manufacturer’s instructions. Sequencing libraries were constructed by using 0.5–1.0 µg RNA per sample. The integrity of total RNA was assessed with the Fragment Analyzer (Agilent) using the DNF-471 RNA Kit (15 nt). Sequencing libraries were generated using the TruSeq Stranded mRNA Library Prep Kit^®^ (Illumina) following manufacturer’s recommendations with PolyA selection for ribosomal RNA depletion. Libraries were sequenced at the sequencing facilities of the University of Valencia (SCSIE-UV) on an Illumina NextSeq 500 platform and 75 bp single-end reads were generated. All raw reads were deposited in SRA-NCBI under BioProject PRJNA1077792 (https://dataview.ncbi.nlm.nih.gov/object/PRJNA1077792?reviewer=a0ou4ekoslho1g9qojfsdm1van).

### Transcriptome assembly and functional annotation

The quality of raw reads was checked using FastQC and MultiQC [25, 26]. Adapters, low-quality and short reads were filtered out using Trimmomatic v.0.39 [27] with the following parameters: SLIDINGWINDOW:4:20 LEADING:3 TRAILING:3 MINLEN:30. Cleaned reads were *de novo* assembled into transcripts using Trinity v2.12 [28] with default parameters. The initial assembly was then processed using EvidentialGene tr2aacds pipeline 2022.01.20 [29], which provides the subset of most accurate coding genes and alternative transcripts. Quality statistics of the assembly were obtained with TrinityScan, and completeness of the transcriptome was assessed with BUSCO 5.4.5 [30] using Viridiplantae_Odb10 and Embryophyta_Odb10 databases.

Transcripts were functionally annotated by sequence homology search using BlastP against UniProt TrEMBL, Swissprot and *Arabidopsis thaliana* genome (Araport 11) (all databases downloaded 2023-03-03). Functional descriptions were processed and summarized using AHRD (https://github.com/groupschoof/AHRD) with weights of 100, 90 and 30 for SwissProt, Araport11 and TrEMBL annotation respectively. Gene ontology (GO) annotations were retrieved from the European Bioinformatic Institute GO annotation database (GOA) (downloaded 2023-03-16) based on the best Blast match against UniProt. Protein domain homology was obtained with HMMER v.3.3 (http://hmmer.org) against Pfam-A release 35 [31], and structural information of signal peptides and transmembrane motifs were predicted using Signal-P v 4.1 [32] and tmhmm v.2 [33], respectively. KEGG ontology terms were assigned to transcripts using BLastKOALA [34]. Additionally, homology searches using BlastP against the *Pinus tadea* reference genome [35] and the ProCoGen assembly of *Pinus pinaster* [36] were conducted.

### Differential expression analysis

The transcriptome assembly served as the reference for read mapping. Cleaned reads from individual samples were mapped to the reference using Bowtie2 [37], using the “sensitive” mode and allowing a maximum of 2 mismatches and a 0.1 mismatch rate. Read counts for each transcript were quantified using RSEM v1.3.1 [38]. Counts were normalized using a trimmed mean of M-values (TMM) using the R package *edgeR* [39]. Expression values were explored using a generalized principal component analysis (GLM-PCA) using the R package *glmpca* and a hierarchical clustering based on the Pearson Correlation distance of the 1000 most variable genes. Both analyses were performed using the R functions ‘prcomp’ and ‘heatmap.2’ of the package *gplots*. Differentially expressed genes were detected using a linear model with genotype and temperature treatment as factors, using the GLM approach in the R package *edgeR*. Additionally, statistical contrasts among pairs of temperature treatments were performed (18 vs. 23 ˚C, 23 vs. 28 ˚C and 18 vs. 28 ˚C). Genes with a false discovery rate (FDR) ≤ 0.05 were considered as differentially expressed genes (DEGs). To specifically study the genes highly up- or downregulated, only those with an absolute Log2 fold-change ≥ 2 (Log2-FC) adjusted *P* ≤ 0.01 were considered. GO enrichment analysis and KEGG enrichment analysis of differentially expressed genes were performed with the R packages *topGO* [40] and *clusterProfiler* [41].

We performed three independent BLAST searches against the *de novo* maritime pine transcriptome, in order to find sets of genes of interest that could be differentially expressed. First, we found transcripts that matched *P. pinaster* genes previously described to be involved in embryogenesis regulation [42], such as *LEAFY COTYLEDON (LEC1)*,* BABYBOOM (BBM)*, and *SOMATIC EMBRYOGENESIS RECEPTOR-like KINASE (SERK)*, as well as genes from the *WUSCHEL-related HOMEOBOX (WOX)* family and from the homeobox transcription factor *(KNOX)* family [43, 44]. Second, a list of genes related with epigenetic regulation was built based on their putative importance from literature review [23, 45]. Additionally, we searched for genes in *Arabidopsis thaliana* annotated with the GO term GO0006306 (DNA methylation), which includes positive and negative regulation of DNA methylation and DNA-methyltransferase activity, and searched against the de novo transcriptome in order to find the orthologs in *P. pinaster*. Third, we searched among DEGs up-regulated in samples from the 28 ˚C treatment for genes related with cell response to abiotic stress, and phytohormones, terpenes or phenols metabolism.

### Validation of DEGs

Differential expression rates estimated for 25 genes were validated by real-time PCR. For this, RNA samples were used for synthesising cDNA using the PrimeScriptTM RT Reagent Kit (Perfect Real Time, Takara Bio Inc.), following manufacturer’s instructions. Quantitative reverse-transcription PCR amplifications were performed in a StepOne Plus (Applied Biosystems, CA, USA), using a final volume of 20 µL containing 0.3 µM of each primer and 10 µL of SYBR Green I Master mix (Takara Bio Inc.) in triplicate for each sample. Amplification conditions were 10 min at 95 ˚C, and 40 cycles of 15 s at 95 ˚C and 60 s at 55 ˚C. Relative expression of each gene was calculated by the ΔΔCt method (Ct = threshold cycle) using the gene coding for Histone 3 as the reference [22]. Genes and primer details are given in Supplementary Table 1.

### Statistical analyses

Gene expression results were subjected to ANOVA when data showed a normal distribution, and mean comparison was performed by Tukey-b post-hoc test. Non-parametric Kruskal-Wallis ANOVA was used when data did not show a normal distribution. All tests were performed using SPSS v25 software (IBM Statistics, USA).

## Results

Variation in maturation temperature did not affect the developmental stage of *P. pinaster* somatic embryos, being most of them at the precotyledonary stage (Supplementary Fig. 1) at the time of sampling. Therefore, the generated transcriptomes (Table 1) corresponded to embryogenic tissues with somatic embryos at the precotyledonary stage, and the altered genetic expression patterns observed are likely temperature-specific adaptive changes. On average, 35 million raw reads were obtained for each library. After quality filtering, the *de novo* assembly produced a transcriptome comprising a total of 57,054 transcripts, which corresponded to 37,919 genes (named as *Ppinas*) with a N50 of 1,881 bp for all transcripts and an N50 for the 90% of the most expressed (Ex90N50) of 2,418 bp. Transcriptome completeness, assessed with BUSCO, showed that 90.6% (62.4% as single copy and 28.2% duplicated) of the 1,614 genes included in embryophyta_odb10 were present, and 3.7% were present as fragmented. On average, 88.0% of the clean reads mapped against the transcriptome (Table 1). Of the gene loci, 24,401 (64%) of them are coding loci with regular-sized proteins, and 13,518 are putative loci with small proteins (smORF < 120 aa). The 27% (10,128) of genes present at least one alternate transcript, with a median of 2 and an average of 2.8 transcripts per locus, reaching a maximum of 24 transcripts. From the 37,919 coding genes detected, 22,736 (60%) have complete proteins and 15,183 have partial proteins. After functional annotation, 16,264 genes were assigned to KEGG terms and 36,710 to GO terms.

**Table 1:** General statistics of number of Raw sequences, clean Reads, and mapped reads against the *de Novo* transcriptome of maritime pine embryonal-suspensor masses maturating at different temperatures

Genotype	Maturation temp. (˚C)	Raw reads	Clean reads	% Clean read (q > 30)	Mapped reads	% Mapped
1058P4c	18	40,150,641	40,146,887	92.7	35,297,813	87.9
	23	37,802,613	37,800,448	92.8	33,259,627	88.0
	28	33,337,959	33,330,015	92.6	29,175,723	87.5
1046P1c	18	37,028,524	37,025,709	93.1	32,252,468	87.1
	23	30,667,851	30,666,528	91.5	27,171,306	88.6
	28	31,028,680	31,023,497	92.4	27,291,159	88.0
1007P3a	18	35,842,315	35,840,580	92.1	31,584,819	88.1
	23	38,929,841	38,927,801	92.4	34,447,670	88.5
	28	34,754,864	34,751,970	92.2	30,649,883	88.2

### DEG enrichment

**Fig. 1:**
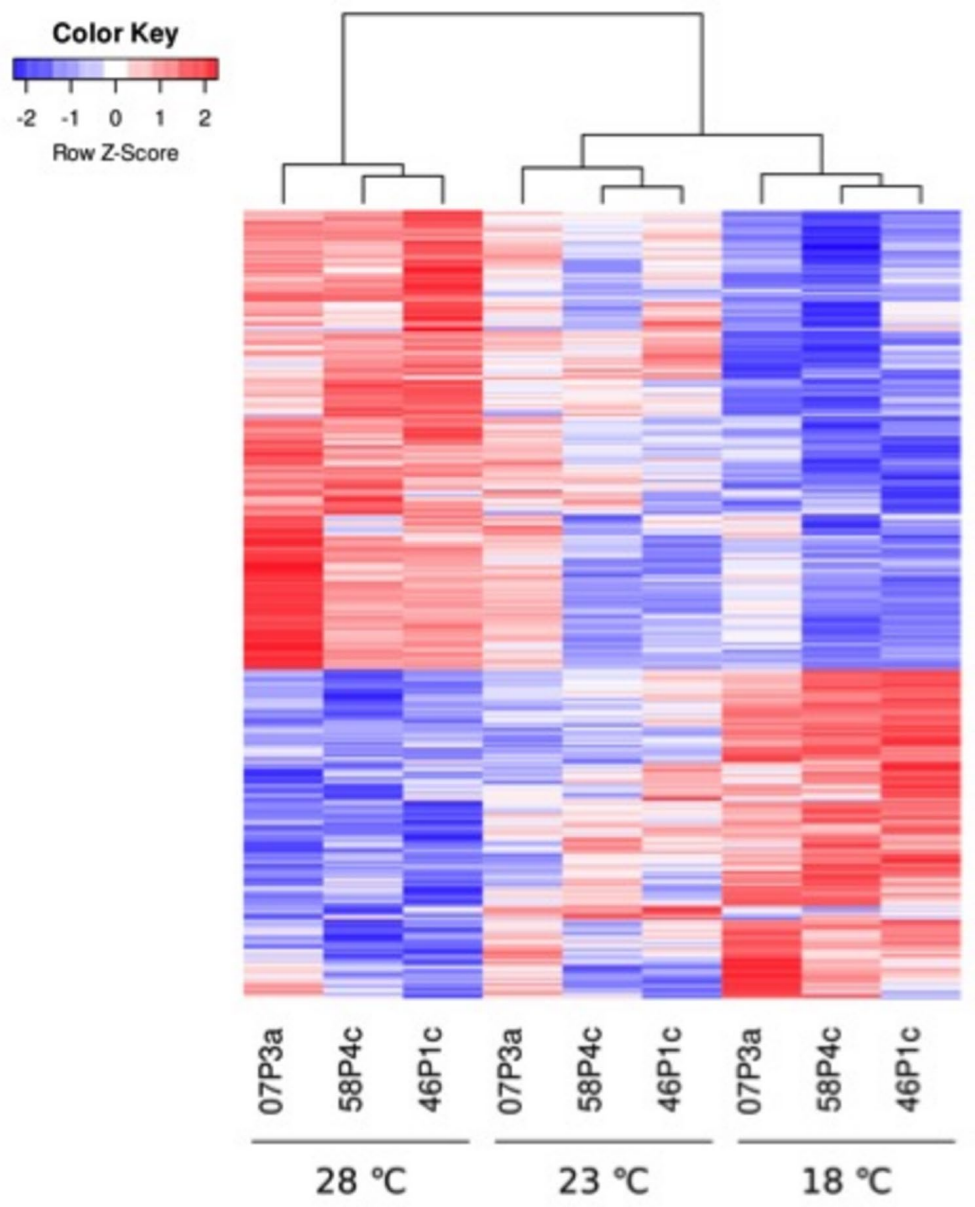
Expression heatmap of maritime pine *(Pinus pinaster)* based on all the differentially expressed genes in embryonal-suspensor masses (EMs) from 3 genotypes (1007P3a, 1058P4c and 1046P1c) maturated at 18, 23 or 28 ˚C

**Fig. 2:**
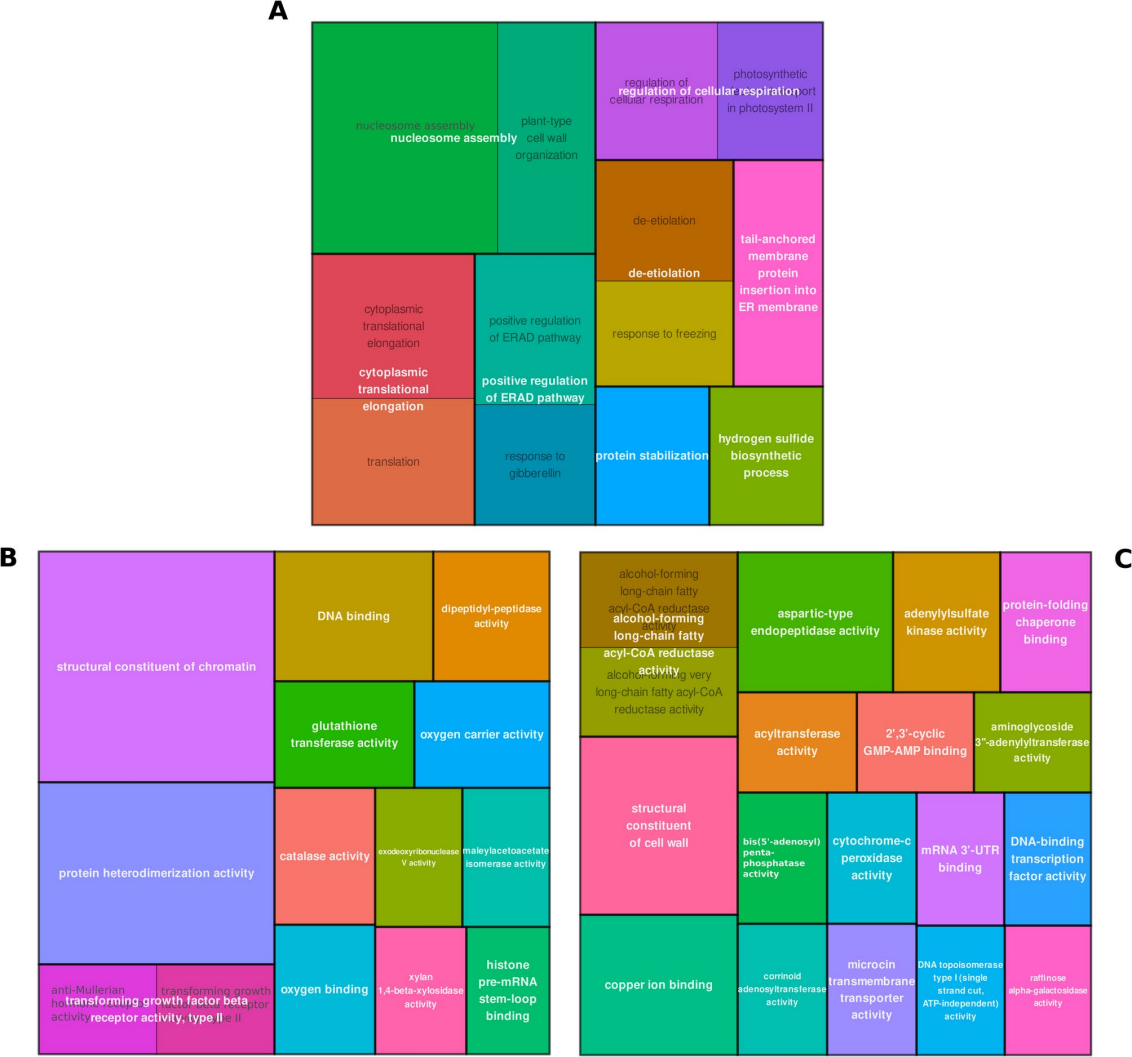
Maps of the GO terms enrichment analysis from 812 DEGs detected among transcriptomes of maritime pine embryonal-suspensor masses (EMs) of 3 genotypes maturated at 18, 23 or 28 ˚C. (**A**) Enriched biological processes; (**B**) molecular functions of DEGs up-regulated (Log2-FC ≥ 2) in EMs of the 18 ˚C epitypes; and (**C**) molecular functions of DEGs up-regulated in the 28 ˚C epitypes

**Fig. 3:**
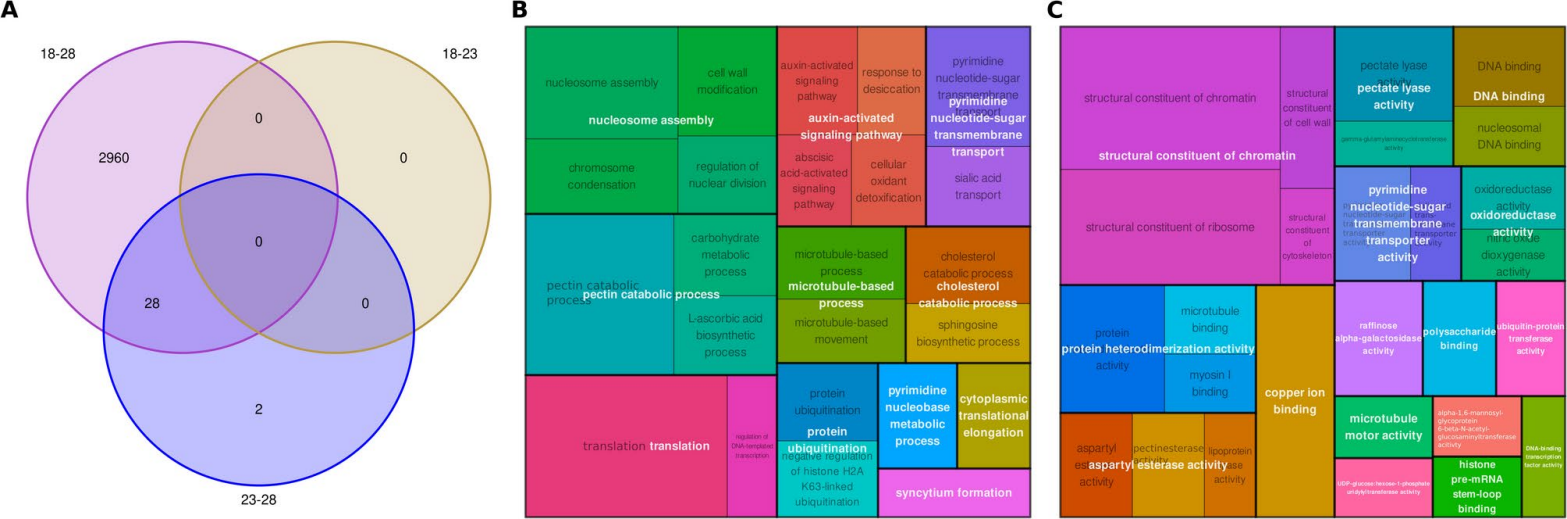
Venn diagram showing the numbers of DEGs detected in pairwise comparisons between the transcriptomes of maritime pine embryonal-suspensor masses (EMs) maturated at 18, 23 or 28 ˚C (**A**). Maps of GO enrichment analysis of the 2,988 DEGs detected between EMs of the 18 and 28 ˚C epitypes: biological processes (**B**), and molecular functions (**C**) of these DEGS

Differential expression analysis identified 812 differentially expressed genes (DEGs) (FDR < 0.05) in response to variation in temperature during somatic embryo maturation (Supplementary Table 2). Differences in the expression profiles could be observed to be affected by the genotype and the maturation temperature (Supplementary Fig. 2). In general, these genes presented opposite expression patterns in EMs maturated at 18 and 28 ˚C, while those maturated at 23 ˚C showed an intermediate response (Fig. 1). Enrichment analysis of GO terms for the 812 DEGs revealed significant enrichment in biological processes associated with nucleosome assembly and protein biosynthesis, stabilization, and degradation, as well as with the regulation of cellular respiration (Fig. 2A). Differentially expressed genes with Log2 FC ≥ 2, that is, highly up-regulated, in EMs of the 18 ˚C epitype encoded proteins with molecular functions related mainly to chromatin structure, protein heterodimerization activity, DNA-binding and oxidative stress response (Fig. 2B). In contrast, DEGs highly up-regulated DEGs in EMs of the 28 ˚C epitype were genes coding for fatty acid metabolism, cell wall structure and protein catabolism (Fig. 2C). The contrast comparing the two more different temperatures (18 vs. 28 ˚C) revealed a total of 2,988 DEGs (Supplementary Table 2, Fig. 3A). The enrichment analysis of this set of genes showed that variation in gene expression between the cold and warm epitypes was related with nucleosome assembly, pectin catabolic process and translation, as well as with auxin-activated signaling pathway, among others (Fig. 3B). These DEGs encoded proteins with molecular functions mainly related with chromatin structure, protein heterodimerization and aspartyl esterase activity (Fig. 3C).

### Effect of temperature on genes regulating embryo development

First, we confirmed that temperature did not affect the maturation stage of our EMs, which could bias the transcriptome profiles, by searching against the *P. pinaster* transcriptome for transcripts annotated as regulators of different embryogenesis phases. We identified three *WOX* genes (*WOX2*, *WOX3* and *WOX13*), four *KNOX* homeobox transcription factors (KN1, KN4, KN5 and KN6), and two *Leafy cotyledon* 1 (*LEC1*) genes, as well as a *BABY BOOM* (*BBM*), and a *SOMATIC EMBRYOGENESIS RECEPTOR-like KINASE* (*SERK*) genes. Four of these genes, *WOX3*,* KN4*, *KN5*, and *KN6* were detected at very low rates. No significant differences among epitypes could be detected for the expression rates of any of these genes (Supplementary Table 3).

### Effect of temperature on genes regulating epigenetic modifications

**Fig. 4:**
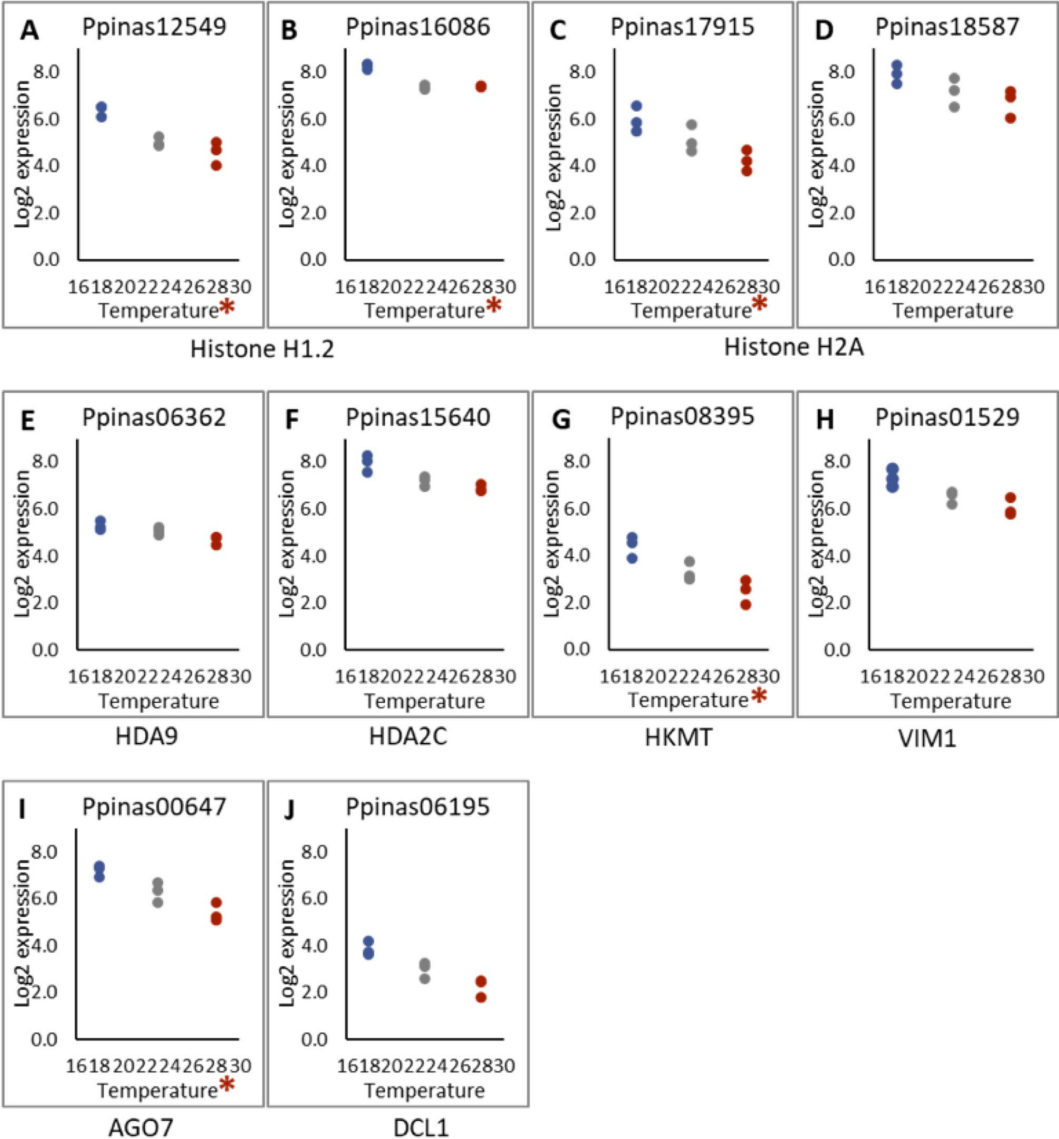
Expression rates (Log2) estimated for 10 *Pinus pinaster* genes annotated as regulators of epigenetic modifications, in 3 EMs (1007P3a, 1046P1c and 1058P4c) maturated at 18, 23 or 28 ˚C. * Denotes significantly different expression rates among the three epitypes, while the remaining genes were differentially expressed between 18 (cold) and 28 ˚C (warm) epitypes

A BLAST search against the maritime pine transcriptome found 37 genes orthologs of Arabidopsis annotated genes related with DNA methylation, and 36 genes previously reported as epigenetic marks regulators in *P. pinaster* genes [23, 45]. This resulted in a set of 61 Ppinas genes potentially involved in epigenetic modifications (Supplementary Table 4). Among them, 10 genes showed differential expression levels between the 18 and 28 ˚C epitypes, being 5 of them differentially expressed also among the three epitypes. Four of these genes encoded for histones: Ppinas12549 (Fig. 4A) and Ppinas16086 (Fig. 4B) coding for *histone H1.2*, and Ppinas17915 (Fig. 4C) and Ppinas18587 (Fig. 4D) coding for *histone H2A*. Two genes encoded for the histone deacetylases HDA9 and HDA2C encoded by Ppinas06362 (Fig. 4E) and Ppinas15640 (Fig. 4F), respectively. Another two genes were related to DNA methylation: Ppinas08395 (Fig. 4G), coding for a histone-lysine methyl-transferase (HKMT), and Ppinas01529 coding for Variant in Methylation 1, VIM1 (Fig. 4H). Finally, two genes involved in post-transcriptional regulation, Ppinas00647 (Fig. 4I), which encodes for an Argonaute (AGO7) protein, and Ppinas06195 (Fig. 4J), which encodes a Dicer-like protein 1 (DCL1), were also found to be differentially expressed in at least two of the three epitypes. The same expression pattern was observed for all these 10 genes: embryogenic lines maturated at lower temperature (18 ˚C) showed higher expression levels as compared to expression rates determined in lines maturated at 28 ˚C.

**Fig. 5:**
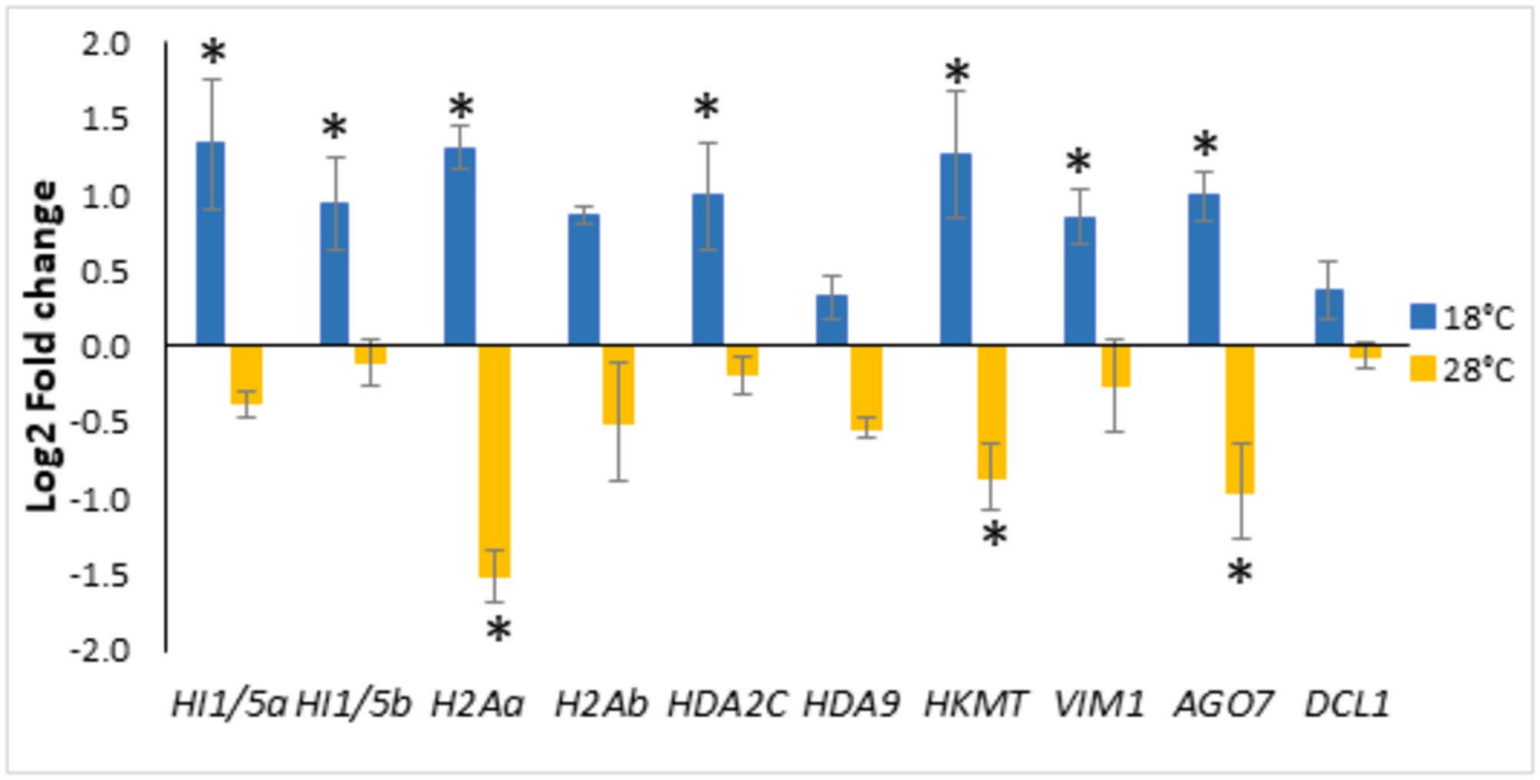
Differential expression rates (Log2-fold-change referred to expression level at control temperature, 23˚C) in maritime pine embryonal-suspensor masses maturated at 18 or 28 ˚C, as determined by qPCR for 10 genes related with epigenetic modulation. From left to right: *HI1/5a* (Ppinas12549), *HI1/5b* (Ppinas16086), *H2Aa* (Ppinas17915), *H2Ab* (Ppinas18587), *HDA2C* (Ppinas15640), *HDA9* (Ppinas06362), *HKMT* (Ppinas08395), *VIM1* (Ppinas01529), *AGO7* (Ppinas00647), and *DCL1* (Ppinas06195). Data are mean ± SE of 3 biological replicates analysed in triplicate. * Denotes significant up/down-regulation (Log2-FC ≥ 1/ Log2-FC ≤ -1)

Amplifications of these 10 genes by real-time PCR corroborated the RNA-seq results, confirming significant differences in their expression levels between embryogenic lines from the cold and warm epitypes. Furthermore, opposite expression ratios were estimated (Fig. 5) in these epitypes referred to standard temperature (23 ˚C). The expression of seven genes was up-regulated (Log2-FC ≥ 1) in embryogenic lines maturated under cold conditions, while three genes were significantly repressed (Log2-FC ≤ -1) in the warm epitype.

### Effect of temperature on genes regulating stress responses

Among the 812 maritime pine DEGs, 277 genes were highly up-regulated in EMs of the 28 ˚C epitype as compared with those of the 18 ˚C epitype. From these, we found 20 genes that also matched specific GO terms (Table 2). Fourteen of these genes matched genes annotated with GO terms associated with cell response to stress (GO:0009737, response to ABA; GO:0009414, response to water deprivation; GO:0009651, response to salt stress; GO:0009408, response to heat; GO:0009409, response to cold). These genes coded for three Ras GTPases-activating protein-binding G3BPs proteins, an aspartic protease, a DNAJ chaperone and an ABA receptor. Four maritime pine transcripts matched genes with GO terms related with phytohormones; GO:0010337, regulation of salicylic acid metabolic process; GO:0019139 cytokinin dehydrogenase activity; GO:0009733, response to auxin; GO:0009695 and jasmonic acid biosynthetic process. Finally, a gene was associated to secondary metabolism (GO:0016102 diterpenoid biosynthesis pathway), and a gene coding for a multicopper oxidase was involved in diphenol activity (GO:0016682).

**Table 2:** List of *Pinus pinaster* genes detected as differentially expressed (Log2 fold change > 2) in embryonal-suspensor masses (EMs) maturating at 28 ˚C as compared to EMs maturating at 18 ˚C, and annotated with GO terms associated to cell response to stress. For each gene the information of KEGG and GO annotation, as well as if it has been checked using qPCR, is shown

Ppinas	Log2-FC	Gene	KEGG	GO Terms	qPCR
19,236	6.2	Ras GTPase-activating protein-binding protein 2 *(G3BP)*	AT5G60980	GO:0009737	+
27,976	5.8				+
14,455	5.5				+
10,458	4.5	RING-type E3 ubiquitin-transferase	AT3G46620	GO:0009737 GO:0009414	
05414	2.3	AAA-ATPase *(AATP)*	AT5G40010	GO:0009737 GO:0009409 GO:0009651 GO:0009414	+
21,581	4.5	*APF2*, Aspartyl protease family protein	AT3G18490	GO:0009737 GO:0009414	+
19,983	2.1	*MBF1*, Multiprotein-bridging factor 1	AT3G24500	GO:0009737 GO:0009408 GO:0009414	+
12,399	2.8	Xyloglucan endotrans-glucosylase/hydrolase (*XTH6*)	AT5G65730	GO:0009414	+
31,854	3.2	*PYL3*- ABA receptor	Osa 4,328,916	GO:0009409 GO:0009414	+
12,480	5.6	Fatty acyl-CoA reductase	AT3G44540	GO:0009651	
19,579	3.9	RING-type E3 ubiquitin-transferase (*R3UT*)	AT2G40830	GO:0009651	+
14,550	2.7	DNAJ-domain containing protein (*DNAJ*)	Tmel_0087	GO:0009408	+
02627	2.0	BAG-family molecular chaperone *(BAG)*	AT2G46240	GO:0009408	+
01805	2.1	*FAO3*, Long-chain-alcohol oxidase	CAP15762	GO:0009409	+
07079	3.3	*OPR*, 12-oxophytodienoic acid reductase	Osa 4,345,762	GO:0009695	+
26,033	5.5	Auxin-responsive protein	AT5G20820	GO:0009733	
25,173	5.6	Peptidase A1 domain-containing protein	AT5G33340	GO:0010337	
11,489	5.5	*CKX5*, Cytokinin dehydrogenase	AT1G75450	GO:0019139	
13,796	4.8	*MPIM1_PINCO*, Monofunctional pimaradiene synthase		GO:0016102	+
23,368	5.6	*LPR1*, Multicopper oxidase	AT1G23010	GO:0016682	+

**Fig. 6:**
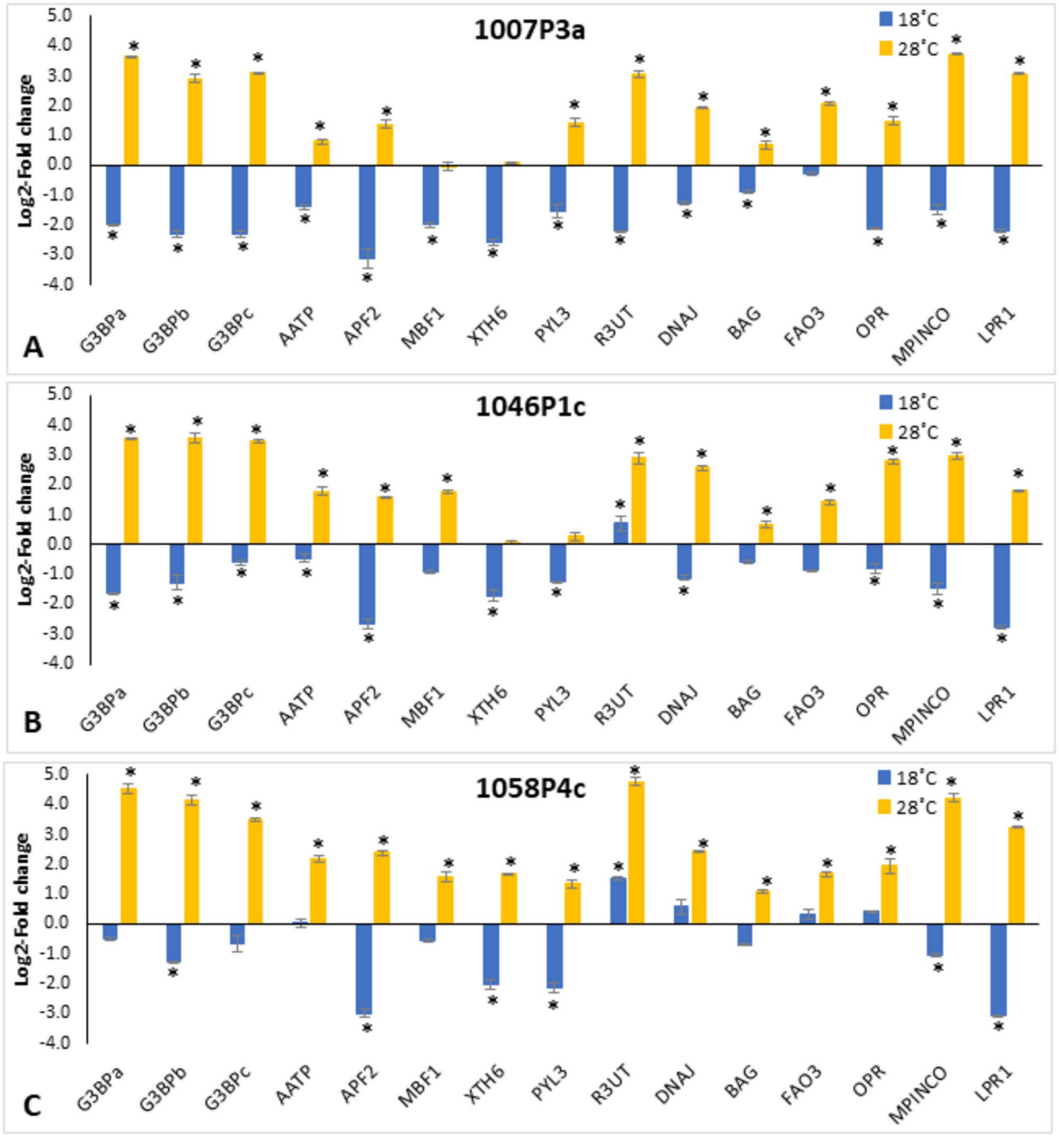
Differential expression rates (Log2-fold-change referred to expression level at control temperature, 23 ˚C) in maritime pine EMs maturated at 18 or 28 ˚C, as determined by qPCR for 15 genes related with abiotic stress response. Data are mean ± SE of 3 replicates performed for 3 genotypes: 1007P3a (**A**), 1046P1c (**B**), and 1058P4c (**C**). * Denotes significant up/down-regulation (Log2-FC ≥ 1/ Log2-FC ≤ -1)

We could validate by real-time PCR the expression rates of 15 of these genes. For all three genotypes, differential expression ratios were observed for these 15 genes in EMs from the cold and/or warm epitypes as compared to the standard temperature condition (23 ˚C), although variation among genotypes was detected. In EMs from the 1007P3a genotype, 13 genes were up-regulated (Log2-FC ≥ 1) at 28 ˚C, and 14 were repressed (Log2-FC ≤ -1) at 18 ˚C (Fig. 6A). In EMs from the 1046P1c genotype, 13 genes were up-regulated in EMs from the warm epitype and 12 were repressed in the cold epitype (Fig. 6B). A gene coding for a RING-type E3 ubiquitin-transferase (Ppinas19579) was, however, up-regulated in both epitypes. Finally, results from the 1058P4c genotype showed that the 15 analyzed genes were up-regulated in EMs of the warm epitype, and only 6 genes were significantly repressed in the cold epitype (Fig. 6C).

Among the genes with the most more contrasting expression levels in EMs between cold and warm epitypes (mean difference in Log2-FC > 4), we found three genes coding for Ras GTPase-activating protein-binding (G3BP) proteins (Ppinas019236, Ppinas027976 and Ppinas014455) and one gene (Ppinas21581) encoding a protein of the aspartyl protease family (APF2), which are associated with ABA and water deprivation response. The *MPIM1_PINCO* gene (Ppinas13796), which is involved in the biosynthesis of pimaradiene, and the *LPR1* gene (Ppinas23368), which encodes a multicopper oxidase involved in phenolic activity, also showed also wider differential expression between epitypes.

Some genes were up-regulated at 28 ˚C in EMs from the three genotypes, such as a gene coding for an AAA-ATPase (Ppinas05414), a gene encoding for a RING-type E3 ubiquitin-transferase (Ppinas19579), and the *FAO3* gene (Ppinas01805), which encodes a long-chain-alcohol oxidase involved in the cell response to cold, and two genes involved in heat response regulation, Ppinas14550 and Ppinas02627, which encode a DNAJ-domain containing protein and a BAG-family protein, respectively. This trend was also observed for the *OPR* gene (Ppinas07079), which encodes a 12-oxophytodienoate reductase involved in the biosynthesis of jasmonic acid. In contrast, three maritime pine genes, Ppinas19983, which encodes a transcription factor MBF1, Ppinas12399, encoding a XYLOGLUCAN ENDOTRANS-GLUCOSYLASE/HYDROLASE (XTH6), and Ppinas31584, which codifies for an ABA receptor-like protein (PYL3) showed different patterns of expression in the three genotypes.

## Discussion

In recent years, an increasing number of studies have identified the genetic and epigenetic molecular components that regulate stress response and plant stress memory [10, 46]. Epigenetic mechanisms represent a set of, often inherited, molecules that modify DNA accessibility to enzymes, thereby influencing gene expression and patterns of mRNA splicing [47].

In conifers, first evidence of stress memory was reported by Johnsen et al. [48] who demonstrated that temperature during zygotic embryogenesis and seed maturation affected climatic adaptation in Norway spruce. Since somatic embryogenesis mimics zygotic embryogenesis, this technology, combined with environmental and chemical stimuli, is increasingly being used to enhance plant defences against abiotic and biotic stresses. This strategy serves to compliment conventional breeding programs. For example, our group has generated *Pinus pinaster* plants with better adaptation to subsequent stress conditions by altering culture conditions or by “priming”, at the somatic embryogenesis induction [19, 20]. Also, in previous work we reported that two-year-old maritime pine plants generated from EMs maturated at 18, 23 or 28 ˚C presented constitutive phenotypic differences and altered response to heat stress [21]. Surprisingly, plants derived from the cold epitype showed better performance when subjected to subsequent heat stress, as demonstrated by its faster and higher proline increase, lower increases in ABA levels, no reduction in active cytokinins, and a better net photosynthesis rate recovery. In turn, plants derived from the warmer epitype displayed higher basal levels of ABA and total cytokinin before heat stress [21]. To study the epigenetic modifications induced in EMs which underlay these altered phenotypes, in the present work we sequenced the transcriptomes of maritime pine EMs from the three epitypes generated by varying the standard temperature during somatic embryo maturation by ± 5 ˚C.

Altering temperatures during the early maturation of somatic embryos led to substantial variation in the transcriptome profile of the cold and warm epitypes (Fig. 1). The transcriptome analysis of maritime pine EMs from the three epitypes identified 812 genes that were differentially expressed (DEGs), exhibiting opposite expression trends across the temperature gradient. Genes up-regulated at low temperature conditions and down-regulated in the warm epitype were functionally annotated mainly in relation with chromatin structure, protein heterodimerization activity, DNA-binding and oxidative stress response (Fig. 2B). Conversely, genes with increasing expression levels as temperature raised -those up-regulated in EMs of the warm epitype and down-regulated in the cold epitype- were related with fatty acid metabolism, cell wall structure and protein catabolism (Fig. 2C).

Our transcriptome analysis identified 10 DEGs related to epigenetic modifications, which were up-regulated in EMs of the cold epitype and down-regulated in those of the warm epitype. The set of epigenetic regulators detected in our study included genes coding for histones, a histone methyltransferase, two histone deacetylases (*HD2C* and *HDA9*), and a Variant in Methylation (VIM1), as well as for 2 genes involved in RNA silencing (*AGO7* and *DCL1*). For seven out of the 10 genes, the results were validated by qPCR (Fig. 5). Histone allelic variants can alter nucleosome properties, leading to changes in nuclear processes such as transcription and epigenetic states, which in turn results in significant modification of plant biology [49]. Our qPCR analysis confirmed that three out the four histone encoding genes- *HI1/5a* (Ppinas12549), *HI1/5b* (Ppinas16086), and *H2Aa* (Ppinas17915)- were up-regulated in EMs maturating at 18 ˚C, with *H2Aa* exhibiting the most pronounced differential expression between epitypes. The expression of the fourth histone, a putative *H2Ab* (Ppinas18587), which contains a histone methylase domain but differs in its C-terminal motif, did not change when compared with control conditions (23 ˚C), but it varied significantly between the two extreme temperatures (Fig. 5). Norway spruce has many variants of histone H2A, which possible roles in epigenetic regulation are still unknown [10], while accumulation of histone H2A was referred to be implicated in heat memory in Monterey pine (*Pinus radiata*) [50]. The differential expression– up-regulation at 18 ˚C and down-regulation at 28 ˚C- of a lysine-methyl-transferase *HKMT* (Ppinas08395) was also confirmed by qPCR. This gene is homologous to ARABIDOPSIS TRITHORAX-RELATED PROTEIN 6 (ATXR6), a plant-specific H3K27 methyltransferase (Supplementary Table 4). In Arabidopsis, ATXR6 plays an integral role in connecting epigenetic silencing with genomic stability [51] and it is also related to cold stress response [52]. Therefore, up-regulation of *HKMT* could be involved in maintaining genome stability during SE maturation at 18 ˚C. These results agree with those referred by Trontin et al. [17], who recently reported that cytosine methylation marks induced by changing temperature during somatic embryogenesis of a maritime pine genotype (PN519), were found preferentially in genes as compared to promoters, and maintained at post-embryonic phases.

With regard to the two histone deacetylases, qPCR confirmed the upregulation of *HDA2C*, which belongs to a plant-specific gene family that is emerging as a crucial player in different aspects of plant growth, development, and in response to different environmental stresses [53]. The other histone deacetylase, *HDA9*, is known to transduce heat signals from the cytoplasm to the nucleus, positively regulating heat responses in Arabidopsis [54], and it has also been reported to repress stress tolerance response by interacting with and regulating the DNA-binding and transcriptional activity of *WRKY53*, a high-hierarchy positive regulator of stress response [55]. However, despite its significant signal in the transcriptome analysis, the differential expression of this gene in the cold and warm epitypes referred to standard temperature conditions could not be validated by qPCR (Fig. 5). The putative *Variant in Methylation 1* (*VIM1*) gene was found to be up-regulated under cold conditions in the qPCR analysis. The *VIM1* gene encodes a methyl cytosine-binding protein that promotes CpG methylation, leading to gene silencing and mediating the memory of epigenetic states [56]. Finally, regarding the genes involved in plant RNA silencing machinery, only a putative *Argonaute 7* gene (AGO7) was confirmed to be up-regulated in EMs at 18 ˚C. The ARGONAUTE family of effector proteins work in sRNA-guided gene silencing pathways, regulating gene expression and transposon activity. The AGOs facilitate cleavage of transcripts, translational repression, RNA-directed DNA methylation, and siRNAs synthesis. These proteins also influence miRNA accumulations, thereby regulating phytohormone signaling pathways and the expression of specific target genes by miRNAs, in a highly stimulus-dependent manner [57].

Previous studies in maritime pine and other conifers have reported the importance of epigenetic regulation on zygotic embryogenesis irrespective of maturation temperature. Rodrigues et al. [23] found higher expression levels at early zygotic embryogenesis for most of the epigenetic regulation-related DEGs, while Vega-Bartol et al. [45] reported that maintenance of chromatin silencing, specific histone modifications, and DNA methylation were co-regulated functions during early zygotic embryogenesis. In our study, performed with somatic embryos at the precotyledonary stage, the higher expression levels of epigenetic regulation-related genes were observed in the cold epitype, but we found altered expression profiles for specific genes, since both histone deacetylase- coding genes *HDA2C* and *HD9* showed the same pattern of expression, while Vega-Bartol et al. [45] referred increasing transcription along embryo development for *HDA9*, as well as for an ortholog of a gene coding for a histone lysine methyltransferase and two putative argonaute (*AGO1* and *AGO9*) genes, while transcription of *HDA2C* and a *Dicer Like 1* (*DCL1*) protein-coding gene decreased towards embryo maturation. A putative *Variant in Methylation 1* (*VIM1*) gene, was also referred to be up-regulated at early and late embryogenesis, being down-regulated specifically at mid embryogenesis. In contrast, Rodrigues et al. [23] found maximum expression of the *VIM1* gene at early embryogenesis that decreased towards mature embryo. Therefore, up-regulation of *HDA9*, *HKMT*, and *AGO7* in EMs of the cold epitype would be low temperature-induced epigenetic marks.

Our results partially align with those reported by Yakovlev et al. [14, 58] who studied temperature-induced differential transcriptomes at early embryogenesis in Norway spruce. They found that the main mechanisms for establishing a temperature-induced epigenetic memory were DNA and histone methylation, and chromatin remodeling, functions that were up-regulated under cold (18 ˚C) conditions as compared to warm (28 ˚C or 30 ˚C) epitype-inducing embryogenesis conditions. In radiata pine, DNA methylation was associated with the establishment of epigenetic stress memory; however, the authors did not address the putative (epi)genetic determinism of such a memory [59]. Conversely, Norway spruce transcriptomic and sequencing studies evidenced that the entire complex of epigenetic regulation may be involved in temperature-induced memory [14, 60]. Furthermore, Yakovlev and Fossdal [60] discovered that this memory was based on differentially expressed sRNA populations that regulate gene expression by modulating the epigenetic machinery of the cell. Therefore, applying low temperatures during somatic embryogenesis in both Norway spruce and maritime pine resulted in increased expression of genes coding for regulators of gene expression that act at both transcriptional and post-transcriptional levels, and that mediate plant developmental processes and stress responses. For instance, histone deacetylation has been linked to gene repression, and both HDAs and AGOs have been recently reported to be involved in heterochromatin stabilization during heat stress [61]. The up regulation of both genes in the cold epitype might be responsible for the heat-tolerant maritime pine phenotype previously reported in the derived somatic plants [21]. We already reported higher expression rates for a *HDA9* gene in heat-primed maritime pine EMs, as well as for a gene coding for a WRKY transcription factor [19]. Interestingly, some expression patterns differed from the results found in Norway spruce epitypes by Yakolev et al. [14]. For example, a DNA methyl transferase (*PaDMT*) and a histone acetyltransferase *(PaNAT1*) were up-regulated in the cold Norway spruce epitype, whereas most of the genes involved in epigenetic regulation, including an argonaute (AGO9) and a histone-lysine-n-methyl transferase (SUVR4) were up-regulated in the warm ecotype. These differences on the transcriptomic profiles of Norway spruce and maritime pine needs further investigation, but we suggest that the geographical distribution of both species, and thereby the habitat temperature, colder for the spruce and warmer for maritime pine, activates epigenetic marks when exposure to contrasting temperatures.

In the warm epitype, an up-regulation of genes associated with abiotic stress responses such as genes involved in water deprivation or ABA signaling pathways was observed. Among them, three genes coding for Ras-GTPase-activating protein-binding proteins (G3BPs) were significantly up-regulated at 28 ˚C (Fig. 6A and B, and 6C). In Arabidopsis, G3BPs have been localized into stress granules upon heat shock. Stress granules are cytoplasmic RNA-protein storage sites formed in response to adverse conditions with mostly translationally inactive mRNAs. When these structures disassemble, mRNAs are released, therefore RasGAP binding proteins coordinate receptor-mediated signal transduction with RNA metabolism [62]. We also observed significant up-regulation of genes coding for protein-processing regulators, such as a RING-type E3 ubiquitin-transferases, and DNAJ and BAG chaperones, which could be related with post-translational regulation of histones, a process that plays key roles in multiple plant development stages and in several abiotic stress responses [63]. For instance, monoubiquitination of histone 2A has been associated with abiotic stress response [46], and chaperone BAG proteins modulate HSP70 activity in animals and plants [64]. High levels of heat-shock proteins (HSPs), which are involved in the epigenetic regulation of heat signaling, were also found in the warm epitype of Norway spruce embryogenic cells [58], a Pp48570 gene coding for a putative HSP protein has recently been described as a candidate gene for postembryonic memory of high temperature in *P. pinaster* [17]. Proteomic analysis of *Pinus radiata* heat-primed embryogenic lines also revealed higher expression levels of proteins involved in direct stress response, such as HSPs and a DNAJ protein homolog, as well as in RNA processing and translation (AGO1D), and in protein folding/unfolding and transport processes [65]. Interestingly, de María et al. [66] referred that two chaperone-coding genes, *HSP70* and *DNAJ*, were constitutively expressed in needles of drought-tolerant *P. pinaster* genotypes.

Two genes encoding for an AAA-ATPase (*AATP*, Ppinas05414) and an aspartic protease (*APF2*, Ppinas21581), which are involved in seed development and germination, were also found to be up-regulated in maritime pine EMs maturating at 28 ˚C. The Arabidopsis AATP1 gene, which encodes a mitochondrial protein, has been observed to be highly expressed during seed maturation, and it is also, ABA-mediated, induced by abiotic stresses [67]. Genes coding for an AAA-ATPase and an aspartic protease have been referred to be involved in maize drought tolerance [68]. Upregulation of some genes involved in biosynthesis of jasmonic acid (JA) (*OPR*, 12-oxophytodienoic acid reductase) and pimaradiene were found in EMs of the warm epitype. Conversely, Trontin et al. [17], suggested that a member of the Cytochrome P450 monooxygenase gene, that blocks the active form of jasmonate (JA-Ile) and was activated during cold-maturation, is a candidate gene for the establishment of epigenetic memory during embryo maturation in maritime pine. Jasmonic acid accumulation was observed in *P. pinaster* seedlings after drought stress, and this hormone was proposed to have a role in the environmental adaptation of the species [69, 70], and also in the defence response against *Fusarium circinatum* [71]. In Arabidopsis, the *ASPG1* (*ASPARTIC PROTEASE IN GUARD CELL 1*) gene has been proposed to function in drought avoidance through ABA signaling in guard cells [72]. Interestingly, some puzzling results were also found. For instance, the expression levels of the ABA receptor *PYL3* and the xyloglucan endotransglucosylase/hydrolase *XTH6* were better explained by down-regulation in cold conditions than by induction in warm conditions (Fig. 6A and B). These were unexpected results, since *XTH6* has been described to be induced by drought stress, and the *PYL3* gene acts as a positive regulator of ABA-mediated inhibition of seed germination, and of tolerance to cold stress. Different *PYL* genes are activated depending on the tissue, the stage of plant development, and the environmental conditions [73]. Up-regulated expression of *PYL3* was reported in rice and Arabidopsis plants exhibiting tolerance to cold and drought stresses [74, 75]. Regulation of core ABA signaling components including PYLs, modulates the sensitivity of tissues to this phytohormone, and is critical to managing excessive and detrimental defence responses to abiotic stress conditions, thus maintaining growth in non-optimal environments [76]. Therefore, the observed lower expression of the *PYL3* gene in maritime pine EMs of the cold epitype might be explained by the need of maintaining embryo development under low temperature conditions. This could account also for the lower expression rates of the *XTH6* gene observed under cold conditions. The *XTH* family genes codify for enzymes responsible of cell wall structural modification and rearrangement, which are involved in a variety of physiological processes in plants, especially in cell elongation and abiotic stress responses. Different expression patterns have been observed for these genes, depending on the tissue and the abiotic stress applied, in several woody plant species such as *Populus trichocarpa* [77], and *Vitis vinifera* [78] subjected to salt or drought stress, respectively, and in somatic embryos of *Dimocarpus longan* subjected to heat stress [79].

Given the above, our results show a clear correlation between the transcriptome of the maturing somatic embryos from the cold epitype and the heat-stress-adapted phenotype of its derived plants, mediated by the up-regulation of genes involved in epigenetics marks. This up-regulation was observed in the 3 EMs lines tested, that were generated from 3 different mother trees. Recently, Trontin et al. [17] reported that cold was a stronger stimulus than heat when inducing temperature memory in maritime pine EMs, and also resulted in more significant phenotypic variations. In contrast, a comparison of the transcriptome profile of the EMs from the warm epitype with the previously reported phenotype of its derived plants with increased ABA and cytokinin content [21] yielded unexpected results. In fact, a gene encoding a cytokinin dehydrogenase, the enzyme that inactivates cytokinin, was up-regulated in this epitype, probably to maintain hormone homeostasis needed for the establishment of the root stem cell niche during embryogenesis [80].

## Conclusions

A number of studies in conifers demonstrated that altering temperature regimes during different somatic embryogenesis steps resulted in phenotypic plasticity in abiotic stress tolerance as a putative memory of temperature conditions.

The present study identifies the transcriptional changes that might activate the epigenetic machinery in maritime pine, and contributes to elucidating the molecular network that coordinate the conifers’ mechanisms of adaptation to non-optimal temperatures. The results support that lowering the maturation temperature during somatic embryogenesis induces epigenetic memory mediated by histones and DNA-methylation marks that modify gene transcription, as well as by posttranslational processes that are maintained in its derived somatic plants. Additional studies regarding the specific genes affected by these epigenetic marks are needed. Further research will also enlighten whether these heat tolerant-induced phenotypes are long-term maintained. Then, our approach would provide a stable molecular basis to increase the adaptive potential of this conifer tree, which is crucial in the context of global climate change.

## Electronic supplementary material

Below is the link to the electronic supplementary material.

Supplementary Material 1

Supplementary Material 2

Supplementary Material 3

Supplementary Material 4

Supplementary Material 5

Supplementary Material 6

## Data Availability

All raw reads were deposited in SRA-NCBI under BioProject PRJNA1077792 (https://dataview.ncbi.nlm.nih.gov/object/PRJNA1077792?reviewer=a0ou4ekoslho1g9qojfsdm1van).
