# Supplementary figures and images for: Temperature-induced variation in the transcriptome of maritime pine (Pinus pinaster Ait.) embryogenic masses modulates the phenotype of the derived plants
Source: BMC Genomics. 2025 May 10;26:467. doi: 10.1186/s12864-025-11610-0 (PMC12065292; doi:10.1186/s12864-025-11610-0)

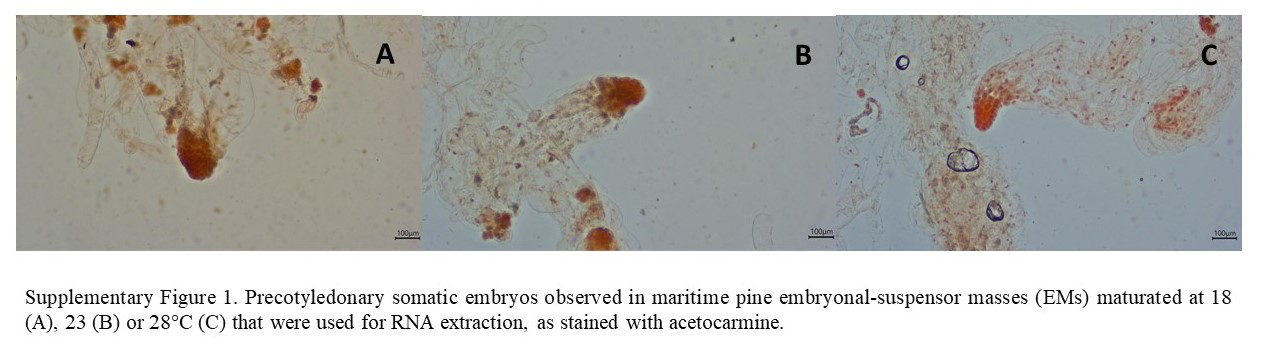

Supplement: Supplementary file 1 — Supplementary Material 1 [file 12864_2025_11610_MOESM1_ESM.tiff]

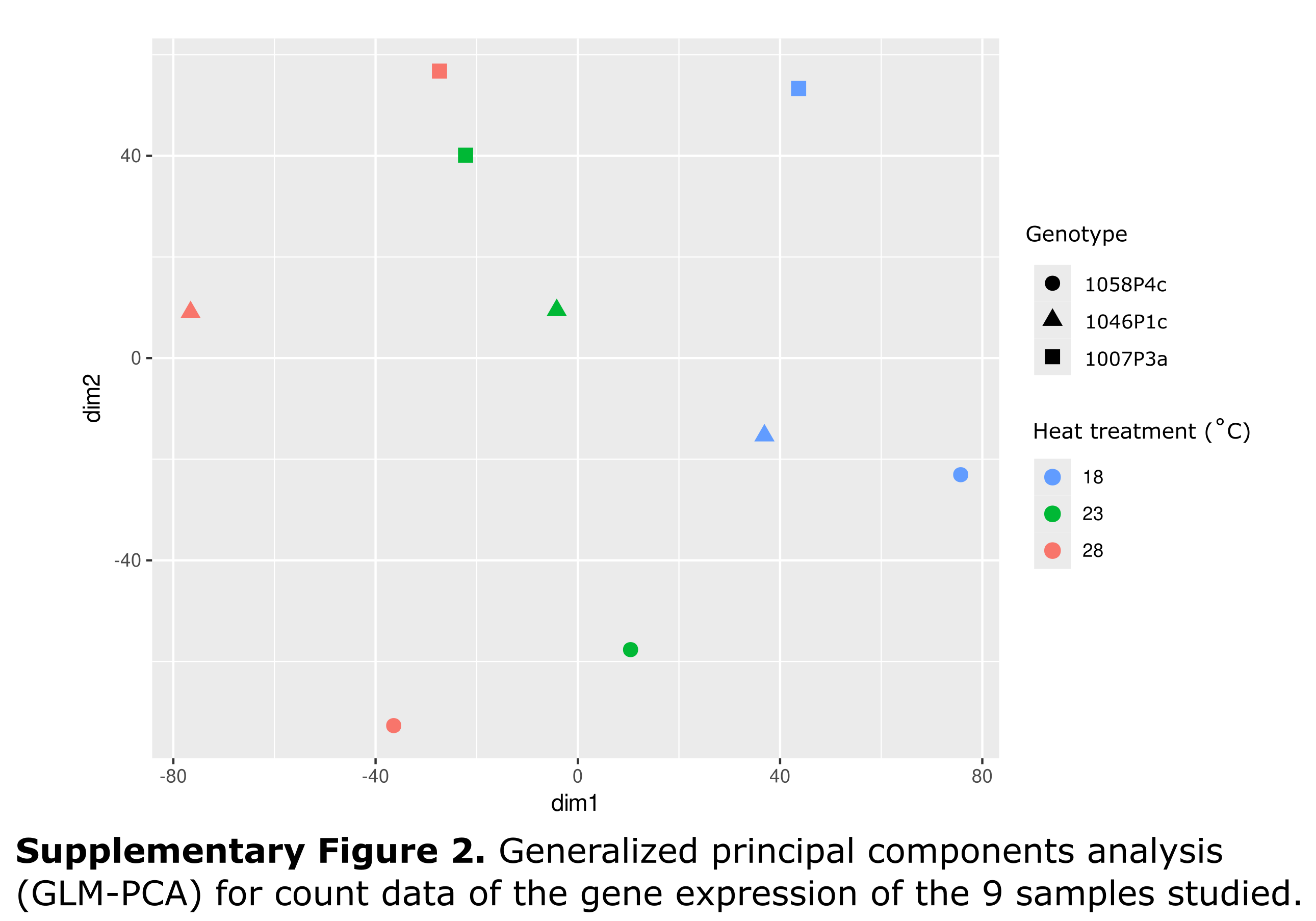

Supplement: Supplementary file 6 — Supplementary Material 6 [file 12864_2025_11610_MOESM6_ESM.tiff]
