# Supplementary material for: Temperature-induced variation in the transcriptome of maritime pine (Pinus pinaster Ait.) embryogenic masses modulates the phenotype of the derived plants
Source: BMC Genomics. 2025 May 10;26:467. doi: 10.1186/s12864-025-11610-0 (PMC12065292; doi:10.1186/s12864-025-11610-0)
Supplement: Supplementary file 2 — Supplementary Material 2 [file 12864_2025_11610_MOESM2_ESM.docx]

**Supplementary Table 3.** Expression rates in 3 epitypes of maritime pine embryonal-suspensor masses (incubated at 18˚C, 23 ˚C and 28 ˚C during maturation) of genes involved in somatic embryogenesis regulation. Data are mean of Log2- of counts per million (CPM) normalized by TMM ± SD of 3 biological replicates.

| ***Gene*** | | **GenBank accession/**  **Sustainpine unigene** | **Ppinas gene** | **Expression rates** | | |
| --- | --- | --- | --- | --- | --- | --- |
|  |  |  |  | **18 ˚C** | **23 ˚C** | **28 ˚C** |
| *WUSCHEL homeobox genes* | *WOX2* | KU962991.1 | Ppinas19391 | 8.40 ± 0.68 | 8.50 ± 0.68 | 8.29 ± 1.00 |
|  | *WOX3* | KU962992.1 | Ppinas19711 | -0.12 ± 0.39 | -0.89± 0.81 | -0.87 ± 0.86 |
|  | *WOX13* | KU962994.1 | Ppinas08417 | 3.04 ± 0.13 | 2.60 ± 0.29 | 2.89 ± 0.23 |
| *KNOX homeobox transcription factor* | *KN1* | KT356208.1 | Ppinas06448 | 3.91 ± 0.30 | 3.37 ± 0.65 | 3.21 ± 0.83 |
|  | *KN2* | KT356209.1 |  |  |  |  |
|  | *KN4* | KT356210.1 | Ppinas23993 | -0.31 ± 0.97 | -0.10 ± 0.95 | -1.10 ± 0.86 |
|  |  |  | Ppinas16045 | -2.69 ± 0.7 | -2.75 ± 1.06 | -2.83 ± 1.00 |
|  |  |  | Ppinas19192 | -2.24 ± 0.6 | -2.75 ± 0.04 | -2.78 ± 0.06 |
|  |  |  | Ppinas29071 | -2.99 ± 0.39 | -2.36 ± 0.41 | -3.04 ± 0.82 |
|  | *KN5* | MK580155.1 | Ppinas11435 | 1.16 ± 0.45 | -0.02 ± 0.61 | -0.21 ± 0.85 |
|  | *KN6* | MK580156.1 | Ppinas10648 | 1.23 ± 1.24 | 1.81 ± 0.63 | 1.92 ± 0.46 |
| *BABY BOOM* | *BBM* | sp_v3.0_unigene12802 | Ppinas01681 | 7.36 ± 0.12 | 7.62 ± 0.18 | 7.56 ± 0.15 |
| *Leafy cotyledon 1* | *LEC1* | sp_v2.0_unigene18226 | Ppinas16143 | 4.13 ± 1.21 | 3.16 ± 0.71 | 3.07 ± 0.69 |
|  |  | sp_v2.0_unigene23255 | Ppinas14178 | 3.09 ± 2.07 | 1.57 ± 1.27 | 1.63 ± 1.24 |
| *SOMATIC EMBRYOGENESIS RECEPTOR-like KINASE* | *SERK* | sp_v3.0_unigene8498 | Ppinas02942 | 7.27 ± 0.32 | 7.50 ± 0.19 | 7.50 ± 0.18 |
