# Supplementary material for: Temperature-induced variation in the transcriptome of maritime pine (Pinus pinaster Ait.) embryogenic masses modulates the phenotype of the derived plants
Source: BMC Genomics. 2025 May 10;26:467. doi: 10.1186/s12864-025-11610-0 (PMC12065292; doi:10.1186/s12864-025-11610-0)
Supplement: Supplementary file 3 — Supplementary Material 3 [file 12864_2025_11610_MOESM3_ESM.docx]

**Supplementary Table 1**. Primers used for qPCR validation of 25 DEGs in maritime pine embryonal-suspensor masses incubated at different temperatures during maturation.

| **Ppinas** | **Gene** | **PRIMERS** | |
| --- | --- | --- | --- |
|  |  | **Forward** | **Reverse** |
| *Genes regulating epigenetic modifications* | | | |
| 00647 | *AGO7* | CCGAGCTGACTTACACCATTTA | GTAGCTCCCGGAGAACAATATC |
| 01529 | *VIM1* | CCCGAATGTTAGTGAGGATGAG | GTTTGGCCTCCCGATTAGAA |
| 06195 | *DCL1* | GAATCCAAATCCTTCCGTTGTG | CCACACTCGATCTGAGGAATAC |
| 06362 | *HDA9* | GGAAATGCCTCCGAAAGATAGA | CACAGTCGATGTGGCTTCATA |
| 08395 | *HKMT* | TGGAGACCCATCTACAACTCT | CCTTCCGGTGTGTGGTTATT |
| 12549 | *HI1a* | CACGGAGGCGATTACTTCTT | CTTGAAATTGGCAGGGAGTTG |
| 15640 | *HDA2C* | TGCTGACAAAGGCAAGAAAACA | CAAGTGGAGTTTCTTTGCCCTT |
| 16086 | *HI1b* | GCCACCTTCTCATCCTACATATC | TAACCTGCGATGGCATACTG |
| 17915 | *H2Aa* | CCCAGTTTCTCGCTCCTTAC | AGAACGGCTGCACCATATAC |
| 18587 | *H2Ab* | GTATCAGAGTGGGAGCAACTG | CGTTTCGCCTTCAGTTCTTTG |
| *Genes overexpressed at 28°C related with abiotic stress, phytohormones, terpenes or phenols* | | | |
| 01805 | *FAO3* | CTTCCCACTAACGACGGTAAAG | GGATAGAGGCAGACCAGTTTATG |
| 02627 | *BAG* | CTGTGGCACGAGAGCTTATT | GCAGTTTCTCTGATGTCTTCCT |
| 05414 | *AAD* | GCACAGGGTCATAGGGATAATG | GTCTGCACTGCCTCGTAAA |
| 07079 | *OPR* | TACATATGAAGGGCGGCATAAT | TCGACGAAACGGGTAAATCTC |
| 12399 | *XTH* | CCTTCGTCGCCTCTTACATATC | CTTCCAAATTCCGGTTGTTACC |
| 13796 | *PMIM* | TGACGTGCTCGGTTACATTAG | CAAATTCAGCTTCCGACCATTC |
| 14455 | *GBPc* | CCCAAAGAGTCCTATGCTTCAA | CCTGGTTCTGGTTATGGTTCTC |
| 14550 | *DNAJ* | TCAGAACTTAGGGCTGCTTAC | CTTAGACTCCTCTGCTACCTTTG |
| 19236 | *GBPa* | GTGCGTTAAATGTGGAGATCAAG | CCACTTACACAAACTATTACTCCATTG |
| 19579 | *R3UT* | CCACAAGGACTGTATCGTTGAT | AGGAGATGGTGGAGGAGAATAG |
| 19983 | *MBF1* | GCGTGGAGTGCAAGATTAAAG | TGAGGACGCTTGTGGATAAC |
| 21581 | *APF2* | GCTCTAGACCAGTGCTGATTAC | ACACTCACCTAAATTCCCTTCC |
| 23368 | *LPR1* | CTTCCATCAGGGCCAGAATATG | GCCGGTGGAGTTCATGTAAA |
| 27976 | *GBPb* | CTGCAGATGGAAACGGTAAATG | CTGGCCTACTCCTAACTTGAAC |
| 31854 | *PYL* | AGTGTCAAGGTCGAAGGAAAG | CAAGGTGAATGTTTGGGAACTG |
|  | *HIS3** | GCTGAGGCTTACCTTGTG | CCAGTTGTATATCCTTAGGCATAA |

(*Vega-Bartol et al. 2013a)
